# Sleep Deprivation Influences Diurnal Variation of Human Time Perception with Prefrontal Activity Change: A Functional Near-Infrared Spectroscopy Study

**DOI:** 10.1371/journal.pone.0008395

**Published:** 2010-01-01

**Authors:** Takahiro Soshi, Kenichi Kuriyama, Sayaka Aritake, Minori Enomoto, Akiko Hida, Miyuki Tamura, Yoshiharu Kim, Kazuo Mishima

**Affiliations:** 1 Department of Psychophysiology, National Center of Neurology and Psychiatry, National Institute of Mental Health, Tokyo, Japan; 2 Department of Adult Mental Health, National Center of Neurology and Psychiatry, National Institute of Mental Health, Tokyo, Japan; University of Sydney, Australia

## Abstract

Human short-time perception shows diurnal variation. In general, short-time perception fluctuates in parallel with circadian clock parameters, while diurnal variation seems to be modulated by sleep deprivation per se. Functional imaging studies have reported that short-time perception recruits a neural network that includes subcortical structures, as well as cortical areas involving the prefrontal cortex (PFC). It has also been reported that the PFC is vulnerable to sleep deprivation, which has an influence on various cognitive functions. The present study is aimed at elucidating the influence of PFC vulnerability to sleep deprivation on short-time perception, using the optical imaging technique of functional near-infrared spectroscopy. Eighteen participants performed 10-s time production tasks before (at 21:00) and after (at 09:00) experimental nights both in sleep-controlled and sleep-deprived conditions in a 4-day laboratory-based crossover study. Compared to the sleep-controlled condition, one-night sleep deprivation induced a significant reduction in the produced time simultaneous with an increased hemodynamic response in the left PFC at 09:00. These results suggest that activation of the left PFC, which possibly reflects functional compensation under a sleep-deprived condition, is associated with alteration of short-time perception.

## Introduction

Temporal perception is fundamental to environmental adaptation. Developing time management skills enables us not only to avoid life-threatening situations, but also to gain rewards and establish motor and cognitive skills. Higher organisms have at least two endogenous clock systems [1], [2]. One of these is a circadian pacemaker located in the suprachiasmatic nucleus of the hypothalamus [3], [4] which is driven by a self-sustaining oscillator with a period of about 24 h and provides the time of day as the hour hand of the clock [5]. Another is a stopwatch-like system which perceives brief temporal intervals as the minute hand of the clock [1], [6].

Most studies in human time perception refer to the issue that time perception is sensitive to the length of duration perceived, where the perception of a longer interval with a range of several minutes to hours (long-time perception) [7] shows different properties from the perception of a shorter interval with a range of seconds or within a minute (short-time perception), as well as from the perception of a sub-second interval [8]–[10]. Short-time perception robustly reflects the rate of the stopwatch-like system [8], [11], [12], being shortened or prolonged when the system “speeds up” or “slows down”, respectively [13]. When the stopwatch-like system speeds up, the duration of events may be felt as more extended than the real interval: That may equal to “time expansion”, which enables us to deal with more things or to perform more rapidly.

Neural correlates of the stopwatch-like system are suspected to include subcortical (cerebellum and basal ganglia) structures in addition to the right prefrontal cortex (PFC) [8], [9], [14], [15]. A three-stage model of temporal processing proposed by Pouthas et al. [9] comprises a first clock stage in which an endogenous clock system measures primitive timing intervals, a second storage stage in which measuring time is stored in short-term working memory and a third decision stage in which temporal judgment is finally made in reference to short- and long-term temporal memories. A similar model of temporal processing has been proposed by other researchers [16]–[18], and consensus has almost been reached that the cerebellum and the basal ganglia may contribute to a basic timing process that corresponds to the first stage of the three-stage model [19], [20]. The right PFC, on the other hand, is implicated in making judgments with working memory or attentional processes contributing to temporal processing [9], [21]–[23], although some researchers regard right PFC activity as constituting temporal processing per se [24], [25].

Biological rhythm studies have reported that short-time perception driven by the stopwatch-like system is not independent of the influence of the circadian pacemaker; short-time perception correlates with circadian markers such as core body temperature and melatonin, showing diurnal variation in consequence [7], [13], [26]–[28]. On the other hand, after sleep deprivation, there is less diurnal variation in short-time perception dissociating with endogenous circadian markers [27], [29], [30]; the biological stopwatch is “sped up” as a result [13].

The present study aims at investigating neural responsibility for attenuation of the diurnal variation in short-time perception after sleep deprivation, utilizing the representative experimental paradigm of the time production (TP) method. The hypothesis is that an attenuated variation of short-time perception is associated with an alteration in PFC activity as a consequence of sleep deprivation. With regard to neural vulnerability to sleep deprivation, the PFC shows complicated hemodynamic or metabolic patterns during cognitive performance after sleep deprivation [31]–[33]. In this state, the PFC shows hypoactivity during working memory or arithmetic tasks [34], [35] but hyperactivity during verbal learning or attention-loaded tasks, as well as verbal working memory tasks [36]–[38]. Although hemodynamic or metabolic responses to sleep deprivation on various cognitive tasks may be influenced by the task difficulty and weight of contribution from PFC activity [37], the neural vulnerability of the PFC to sleep deprivation must have consistent repercussions on cognitive performance. Taken together, the vulnerability of the PFC to sleep deprivation presumably influences short-time perception.

We utilize the brain imaging method of functional near-infrared spectroscopy (fNIRS) for the experimental purpose. fNIRS is a noninvasive optical imaging technique to measure changes of cerebral blood flow and volume through fluctuations in local glucose and oxygen coupled with neural electric activity [39], [40]. It is well suited to sleep deprivation studies because it neither produces high noise levels on scanning nor requires that participants severely restrict their body movement, and thus is unlikely to seriously influence the sleep-deprived condition. It is also suitable for monitoring the participants' condition while they are performing tasks.

## Results

### Sleep Data

Nocturnal sleep duration on the interval day between the first and the second experiments for two different sleep schedules [sleep-controlled (SC) - sleep-deprived (SD), and SD - SC] was not significantly different [SC-SD (8.01±0.96 h) vs. SD-SC (8.70±1.72 h); *t*(1,16) = 1.102, *p* = 0.287]. Sleep duration between the first and the second TP sessions during the participants' stay in the laboratory also did not differ significantly between the two sleep schedules [SC-SD (6.40±0.18 h) vs. SD-SC (6.63±0.11 h); *t*(1,16) = 1.145, *p* = 0.269].

### Time Production Data

**Figure 1:**
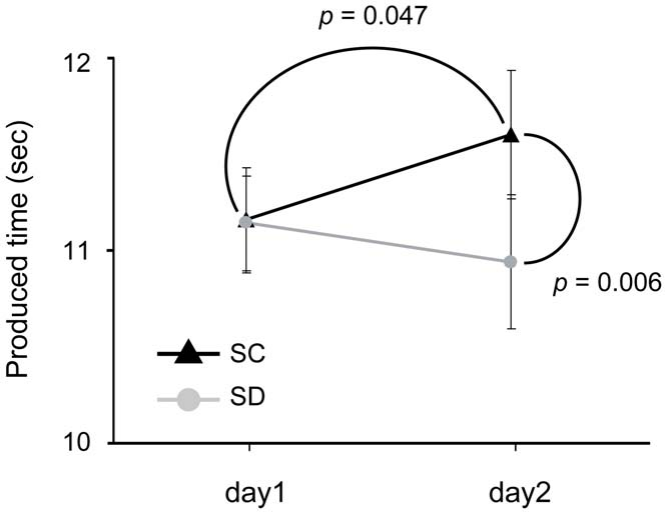
Mean interval variation in SC and SD conditions (n = 18). Black filled triangles with error bars (left: day 1, right: day 2) and the linking line indicate mean produced intervals and the trend of fluctuation in the SC condition, respectively. Gray filled circles with error bars (left: day 1, right: day 2) and the linking line show mean produced intervals and the trend of variation in the SD condition, respectively. Solid curves connecting two markers indicate significant differences (*p*<0.05).

Produced time in the SC condition was 11.16±0.27 s on day 1 and 11.60±0.33 s on day 2, and in the SD condition was 11.14±0.24 s on day 1 and 10.94±0.35 s on day 2 (Fig. 1).

A significant interaction of condition × day was obtained [*F*(1,16) = 9.888, *p* = 0.006]. Pair-wise comparisons of two levels within the factor of day in each condition revealed that the produced time on day 2 was significantly longer than that on day 1 in the SC condition [*F*(1,17) = 4.575, *p* = 0.047]. In the SD condition, on the other hand, the produced time on day 2 was not significantly different from that on day 1 [*F*(1,17) = 0.753, *p* = 0.398]. Produced time on day 2 in the SD condition, relative to that in the SC condition, was significantly attenuated [*F*(1,17) = 6.123, *p* = 0.024].

There was no significant interaction of the between-subject factor of schedule [condition × schedule: *F*(1,16) = 0.254, *p* = 0.621; condition × day × schedule: *F*(1,16) = 0.755, *p* = 0.398]. These findings taken together with those for sleep data indicate that time production performance was not influenced by sleep schedule.

### fNIRS Data

**Figure 2:**
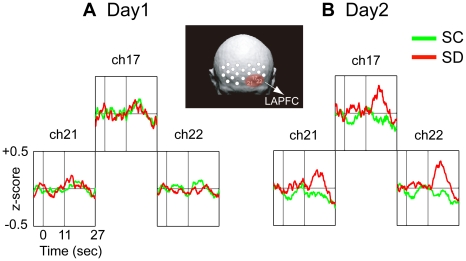
Time course of grand averaged oxygenated hemoglobin (oxy-Hb) concentration changes during 10-s time production trial in the left anterior prefrontal (LAPFC) (n = 14). Comparison of oxy-Hb concentration changes between the SC (green line) and the SD (red line) conditions on (A) day 1 and (B) day 2 in the LAPFC (chs.17, 21, 22). Scores of 0, 11 and 27 below the waveform box indicate the onset of the trial, the end of the trial, and the finish of the post-trial rest, respectively. Oxy-Hb changes are given as z-scores. Any significant differences in oxy-Hb concentration changes between the SC and the SD conditions in the right hemisphere except for in the left hemisphere are not observed on day 2. Marked differences in oxy-Hb concentration changes between conditions are observed in the LAPFC on day 2.

We conducted statistical analyses for fNIRS data utilizing mean values of changes in oxygenated hemoglobin (oxy-Hb) concentration. The overall ANOVA for the midline area did not show any significant difference [condition: *F*(1,13) = 0.322, *p* = 0.580; condition × day: *F*(1,13) = 1.715, *p* = 0.213; condition × channel: *F*(3,39) = 0.458, *p* = 0.713; condition × day × channel: *F*(3,39) = 1.777, *p* = 0.167]. The overall ANOVA for the lateral site showed only a significant interaction for condition × day × hemisphere [*F*(1,13) = 5.373, *p* = 0.037], suggesting that PFC activity in the SD condition, compared with that in the SC condition, was more enhanced in the left hemisphere on day 2 (Fig. 2B). Subsequent planed ANOVAs confirmed that there was a significant difference between conditions on day 2 [condition × hemisphere: *F*(1,26) = 9.049, *p* = 0.006], but not on day 1 [condition × hemisphere: *F*(1,26) = 0.024, *p* = 0.879]. Additional ANOVAs showed that the more enhanced PFC activation in the SD condition on day 2 appeared in the left hemisphere [*F*(1,52) = 6.142, *p* = 0.017], but not in the right hemisphere [*F*(1,52) = 0.225, *p* = 0.637].

**Figure 3:**
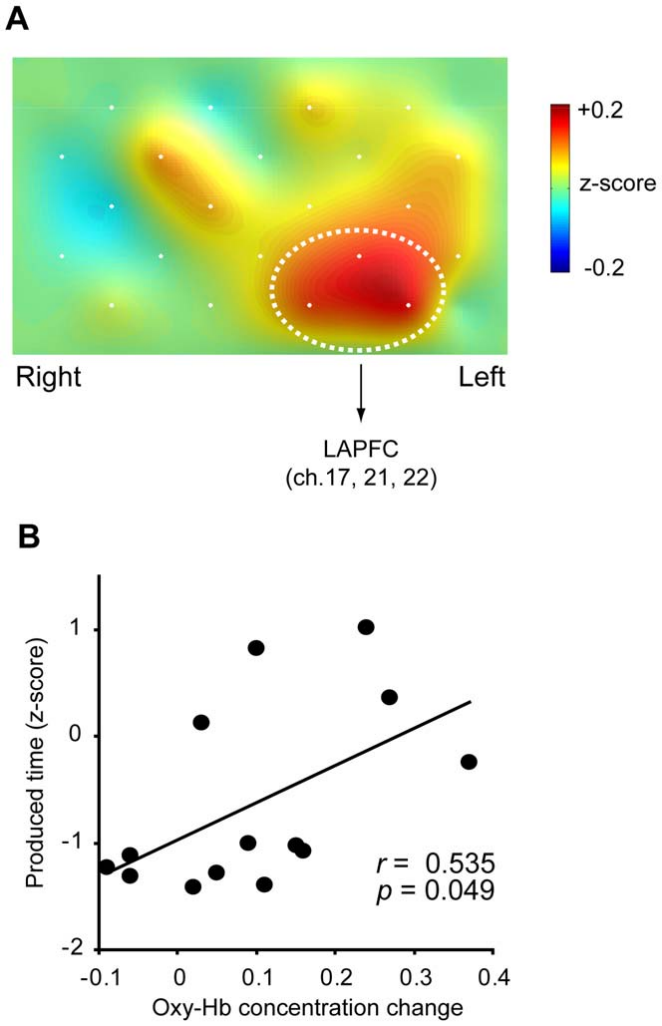
Change in hemodynamic response and functional correlation in prefrontal cortex (PFC) activity after sleep deprivation. (A) Topographic image representing increased activation in the PFC on day 2 in the SD condition (n = 14). The image is constructed by using subtracted values between the SC and the SD conditions (SD minus SC). The area of dotted white circles emphasizes the left anterior PFC (LAPFC) including chs. 17, 21 and 22 for region of interest (ROI) analysis. (B) Correlation between normalized produced intervals and oxy-Hb concentration changes in the LAPFC ROI on day 2 in the SD condition (n = 14). Functional connectivity in changes of short-time perception with LAPFC activity under sustained wakefulness is suggested by a correlation coefficient (*r*) of 0.535 with statistical significance (*p*<0.05).

Enhanced oxy-Hb concentration changes on day 2 in the SD condition, compared with those in the SC condition, were observed in the left anterior PFC (LAPFC) region of interest (ROI) including chs. 17, 21 and 22 (see Fig. 3A). Comparing the mean change in concentration in the SD condition with that in the SC condition, a significant effect was observed in the overall ROI [*t*(1,13) = 2.810, *p* = 0.015 (*p<*0.0375: adjusted α level under the control of the false discovery rate; FDR)]. Each channel in the ROI also showed a significant difference [ch.22: *t*(1,13) = 3.066, *p* = 0.009 (*p<*0.0125: FDR); ch.17: *t*(1,13) = 2.872, *p* = 0.013 (*p<*0.025: FDR)] or trend [ch.21: *t*(1,13) = 2.007, *p* = 0.066 (*p<*0.05: FDR)]. A significant positive correlation was observed between produced time and change in oxy-Hb concentration in the overall ROI on day 2 in the SD condition [*r* = 0.535, *p* = 0.049; Fig. 3B].

## Discussion

Sleep is believed to be a neural state during which both consolidation of memories and homeostatic preservations are taking place [41]–[44]. Sleep deprivation, even for the course of an extended active period of the day, eliminates these effects, and possibly results in a deterioration in cognitive activity [13], [30], [45].

The present study demonstrated that sleep deprivation modulates short-time perception behavior, as was suggested by earlier studies [27], [30]. It has been shown previously that a short-time perception profile exhibits diurnal variation, reaching a peak (the longest produced time) around 09:00 and a nadir (the shortest produced time) around 21:00 with a regular sleep-wake cycle under experimental conditions [13]. Here, we measured produced time around both these expected peak and nadir periods in order to obtain approximate peak-to-peak amplitude of the diurnal variation of short-time perception. As a result, we found that the produced time at 09:00 was significantly attenuated after sleep deprivation compared with that after physiological sleep. It was unlikely that the alteration in properties of time perception after sleep deprivation was caused by circadian phase shifting (different peak times) of short-time perception rhythms between the SC and SD sessions because all participants were exposed to similar time cues such as dim light (<100 lux) and prohibited from physical exercise throughout the experiments, and the produced time levels around 21:00 were in fact identical between the two sessions. This seems to indicate that sleep deprivation per se modulated short-time perception. Given that produced time represents the rate of the biological stopwatch-like system [1], the attenuation of produced time observed on day 2 in the SD condition might be associated with the stopwatch-like system that runs faster [13].

A functional correlation of increased activation of the LAPFC after sleep deprivation with short-time perception behavior was observed in the present study, although unlike in previous studies, we failed to detect any significant changes in right PFC activity [8], [9], [14], [15]. This discrepancy in the localized PFC activation findings between the present and previous studies is possibly attributable to experimental settings, tasks used and imaging modalities [9], [14], [24], [46]. It has also been argued that increased activation of the PFC after sleep deprivation is associated with neural compensation for cognitive function [36], [38], [47], and the LAPFC activity observed in the present study should also be argued in the context of neural compensation: the LAPFC activity likely contributed to the third decision stage of temporal judgment in the three-stage model [9], with adaptive neural activity in the SD condition, although the produced time on day 2 in the SD condition was different from that in the SC condition.

It remains open as to whether or not a subcortical network including the cerebellum and the basal ganglia, assumed to be associated with timing processing, contributes to attenuating the diurnal fluctuation of short-time perception. Sustained wakefulness affects dopaminergic neuronal activity related with cognitive function as well as temporal function [48]–[50]. If alteration of subcortical activities might be caused by sleep deprivation, attenuation of short-time perception probably reflects subcortical vulnerability, and the change in PFC activity is possibly a by-product. Further studies are warranted to elucidate the processes involved.

As has been reported, short-time perception is modulated by affective states [51], an intense fever [52], [53] and psychotic disorders [54], [55]. Diurnal variation of short-time perception associated with the rest-activity cycle is expedient for adapting in daily life, similarly to the vegetative functions in blood circulation [56], aspiration [57], assimilation [58] or incretion [59], [60]. These functions robustly synchronize to diurnal variation oscillated by a circadian pacemaker, and simultaneously prepare for desynchronization from diurnal variation under stressful conditions. Temporarily collapsing the diurnal variation of short-time perception may be an important function for surviving a crisis, such as in a situation with an acute emergency. Time perception in humans, as well as in other organisms, should be fundamentally synchronized to the physical state for constant adaptation to daily lifestyle regularity. However, once we are placed under stress, time perception must desynchronize from regular physical homeostasis and be shortened enabling time expansion and assumedly allowing us to adopt suitable strategies for coping with a stressful environment by doing or thinking of things more rapidly. Serious consideration of an adaptive nature of the human PFC function [61], [62] suggests that the PFC might play a switch-like role in short-time perception as a situation demands, to meet the demands for adaptation.

## Materials and Methods

### Participants

Eighteen healthy right-handed males (mean age 22.4 yr; range 20–28 yr) participated in laboratory-based experiments in a 4-day protocol. A psychiatrist conducted a medical examination to confirm that none of the participants had a history of neurological or psychiatric disorder or abused psychoactive drugs. Participants were asked to avoid intake of caffeine, nicotine and alcohol for one week prior to and during the experiment. For one week before the experiment, they were also required to keep a regular sleep-wake habit in which they slept from around 00:00 and awoke at around 08:00, as confirmed by a sleep log and an activity recorder (Actiwatch-L, Mini-Mitter Co., Inc. Bend, OR). The mean habitual sleep duration was 6.5±0.1 h, and habitual sleep onset and wake times were 00:34±00:13 and 07:22±00:10, respectively. All procedures for the study were carried out in accordance with the principles outlined in the Declaration of Helsinki. The experimental protocol was approved by the ethics committee of the National Center of Neurology and Psychiatry, Japan. All participants gave written informed consent to take part in the study.

### Experimental Protocol

**Figure 4:**
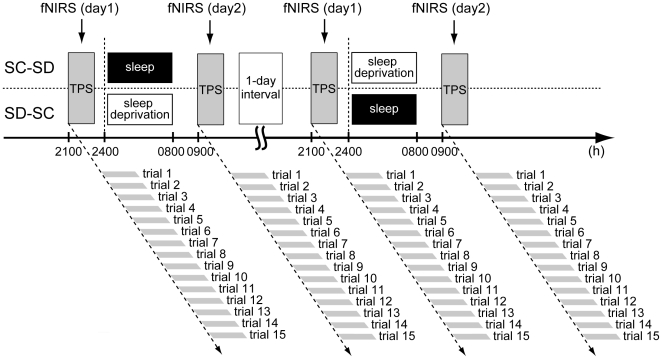
Experimental protocol. Time production sessions (TPS) were conducted twice at around 21:00 on day 1 and about 09:00 on day 2 in the sleep-controlled (SC) and the sleep-deprived (SD) settings. The two conditions were randomly scheduled with a 1-day interval. Each TPS included 15 trials, each of which participants started and ended with their own button presses. Functional near-infrared spectroscopy (fNIRS) data were recorded continuously through the sessions.

All participants performed a 10-s TP task in the SC and SD conditions, scheduled in random order with a 1-day interval (Fig. 4). On the first day (day 1) in both the SC and SD conditions, participants arrived at the laboratory at about 18:00. fNIRS probe and optodes were attached to the surface of the scalp approximately one hour before the start of the TP session (at 20:00). The session, which lasted approximately 15 min, generally started around 21:00–22:00. After the end of the session, in the SC condition, the participants rested without sleep and exercise until 00:00 and then stayed in bed under complete darkness (<0.1 lux) until 08:00 on the second day (day 2). In contrast, in the SD condition, participants were asked to stay awake quietly under room light (100 lux) until 08:00 the next morning. During the nighttime period of enforced wakefulness, researchers monitored the participants' status via a video monitoring system. On day 2, the TP session started again around 09:00–10:00. All the experiments were performed at the time isolation facility of the National Center of Neurology and Psychiatry. The ambient temperature and humidity in the time isolation facility were maintained at 24.0±0.5°C and 60.0±5%, respectively, and temporal information was not provided to participants throughout the experimental schedule.

### Time Production Task

TP tasks were arranged in an event-related design for detecting the hemodynamic response for a single trial. It has been confirmed that output of the task shows diurnal variation correlated with core body temperature or melatonin under a constant condition [7], [13], [26], [28]. It shortens from morning into night, and is prolonged again from night to the next morning [7], [13], [26], [29].On the basis of our previous findings [26], [27], TP sessions in the present study were conducted between 21:00–22:00 on day 1 and 09:00–10:00 on day 2, corresponding to the expected nadir and the peak periods of the diurnal variation of TP in the present participants with a regular sleep-wake cycle, respectively.

Each TP session consisted of 15 trials. The inter-trial interval was about 30 s. Participants were asked to produce a 10-s interval and to begin and end each trial by pressing a key button [13]. Duration from the first to the second button presses was counted as the produced time. Participants were given no feedback about accuracy in the trials.

### fNIRS Data Acquisition

**Figure 5:**
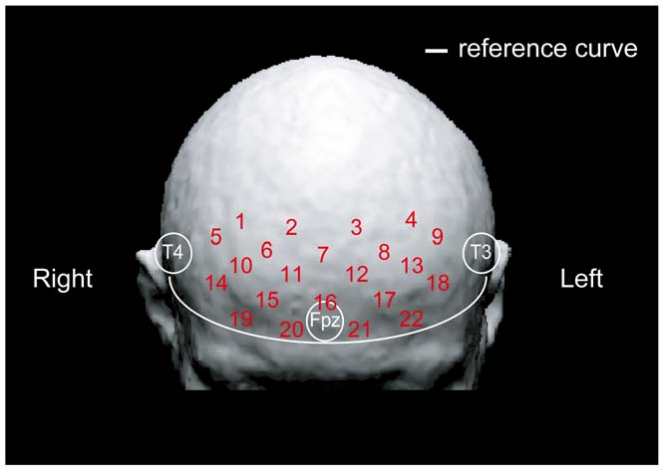
Approximate positions of 22 channels in fNIRS superimposed on the 3D head model. The 22 measuring channels were produced by optodes placed equidistantly over the prefrontal cortex (PFC) area. The lower line of the 3×5 optode probe was positioned along the reference curve linking T3, T4 and Fpz.

Regional hemodynamic changes in brain tissue were monitored throughout the TP sessions by a continuous wave-type fNIRS system (OMM3000; Shimazu Co., Tokyo, Japan), which outputs near-infrared light at three wavelengths (780, 805 and 830 nm). All transmitted intensities of the three wavelengths were recorded every 130 ms at 22 channels in order to estimate concentration changes in oxy-Hb, deoxygenated hemoglobin and total hemoglobin, on the basis of the modified Beer-Lambert equation as a function of light absorbance of Hb and pathlength. A 3×5 optode probe was utilized, in which light detectors and emitters were alternately positioned at an equal distance of 3 cm. Based on the international 10–20 system [63], the lower central edge of each probe was suited above Fpz, along the reference curve of T3 - Fpz - T4. The 22 channels (see Fig. 5) probably covered the middle and superior PFC regions (BA9, 46, 10) [64].

### fNIRS Data Analysis

Oxy-Hb data was chosen to examine event-related response in the PFC since it is an optimal index for changes of regional cerebral blood flow [65]. Data from 4 participants who were recruited to the experiment in the early stage were excluded from fNIRS analyses because they were monitored with a distinct optode probe. We applied a high-pass filter to raw data, re-sampled at 10 Hz, using the low-cutoff frequency of 0.05 Hz. Smoothing was performed by the moving average method (a boxcar filter) with a sliding time window of 1.1 s. Data were converted into z-scores by the normalization process [(*Xi*–*M*)/*SD*; where *Xi* is the value at each time point, *M* is the mean of all points and *SD* is the standard deviation of all points] [66]. Concentration changes time-locked to trial onset were extracted from 5 s before to 27 s after the onset, covering a mean produced time of about 11 s and a mean rest interval of about 16 s. A total of 15 epochs were obtained for each experimental day (day 1 or day 2) in each condition (SC or SD). Before individual averaging, baselines were corrected with mean z-scores of 5 s before trial onset. Grand averaged concentration changes in the LAPFC region, based on statistical analyses, were superimposed (Fig. 2), and subtracted values were utilized for reconstructing a topographic image (Fig. 3A).

### Statistical Analysis

To examine whether the order of the sleep conditions [SC-SD (n = 7) vs. SD-SC (n = 11)] influenced outcomes of time production performance, we compared nocturnal sleep duration in both sleep schedules during the 1-day interval between the first and the second experiments and the sleep duration between the first and the second TP sessions during the participants' stay in the laboratory. We also analyzed the time production data of 18 participants using three-way ANOVA including two within-subject factors of condition (SC and SD) and day (day 1 and day 2), and a between-subject factor of schedule (SC-SD and SD-SC). If significant interactions were obtained, follow-up ANOVAs were conducted.

fNIRS data of 14 participants were tested separately for the lateral and the midline sites by overall ANOVA including within-subject factors of condition, day, hemisphere (left, right) and channel [9 chs: left, 1, 2, 5, 6, 10, 14, 15, 19, 20; right (the corresponding order): 4, 3, 9, 8, 13, 18, 17, 22, 21] for the lateral site, or factors of condition, day and channel (4 chs: 7, 11, 12, 16) for the midline area, utilizing mean z-scores of all temporal points (0–27 s). When the overall ANOVA reached statistical significance of the interaction coefficients, planed ANOVAs were subsequently performed with pooling error terms across the entire analyses, for the purpose to examine our assumption that sleep deprivation modulates the PFC activities on the time production trial. If degrees of freedom exceeded one degree, Greenhouse-Geisser correction was applied on the basis of the sphericity test. Depending on the topographic image reconstructed with subtracted values of changes in oxy-Hb concentration (SD minus SC; Fig. 3A), and a confirmed methodology for fNIRS probe placement [64], we defined the LAPFC, including chs. 17, 21 and 22, which corresponds to the medial dorsal part of the PFC (Brodmann's area 10) [64], as the ROI, and further examined the influence of sleep-deprivation on neural response in the LAPFC. Paired *t*-tests were performed for the ROI and the three constituent channels, with α levels adjusted by Benjamini and Hochberg's method to control the FDR [67]: *H*_(*i*)_ denotes the null hypothesis based on the corresponding *p*-value *P*_(*i*),_ *i* = 1, 2, …, m. *P*_(*i*)_ is increasingly ordered such as *P*_(1)_<*P*_(2)_<…<*P*_(m)_. The FDR control procedure starts with *i* = m, and continues to compare *P*_(*i*)_ with *α*(*i*/m) until *P*_(*i*)_ satisfy the constraint *P*_(*i*)_<or = α(*i*/m). The relation between produced times standardized within each participant and oxy-Hb concentration changes in the ROI on day 2 in the SD condition was tested with Pearson's product-moment correlation coefficient to examine functional connectivity between short TP and neural response of the LAPFC.

Outcomes of the tests for produced time and fNIRS data were considered significant at level α if *p*<0.05. Results are shown as mean and standard errors (SEs). Statistical analysis was performed by SPSS 12.0 J (SPSS Japan Inc., Tokyo).
